# Polysubstituted Isoflavonoids from *Spatholobus suberectus*, *Flemingia macrophylla*, and *Cudrania cochinchinensis*

**DOI:** 10.1007/s13659-017-0121-2

**Published:** 2017-01-21

**Authors:** Li-Xia Wang, Hai-Rong Zheng, Fu-Cai Ren, Tian-Ge Chen, Xiang-Mei Li, Xian-Jun Jiang, Fei Wang

**Affiliations:** BioBioPha Co., Ltd., Kunming, 650201 People’s Republic of China

**Keywords:** *Spatholobus suberectus*, *Flemingia macrophylla*, *Cudrania cochinchinensis*, Isoflavan, Isoflavone, Cytotoxicity

## Abstract

**Abstract:**

Four hitherto unknown polysubstituted isoflavonoids, including three isoflavans: 7,4′-dihydroxy-8,2′,3′-trimethoxyisoflavan (**1**), 7,2′,4′-trihydroxy-8,3′-dimethoxyisoflavan (**2**), and 7,2′,4′-trihydroxy-5-methoxyisoflavan (**3**), and one prenylated isoflavone cudraisoflavone M (**4**) were isolated from the ethanol extracts of *Spatholobus suberectus* (for **1** and **2**), *Flemingia macrophylla* (for **3**), and *Cudrania cochinchinensis* (for **4**), respectively. Their structures were established on the basis of extensive spectroscopic analysis. Compounds **1** and **4** exhibited weak cytotoxic activity against five human cancer cell lines (HL-60, A-549, SMMC-7721, MCF-7, and SW-480).

**Graphical Abstract:**

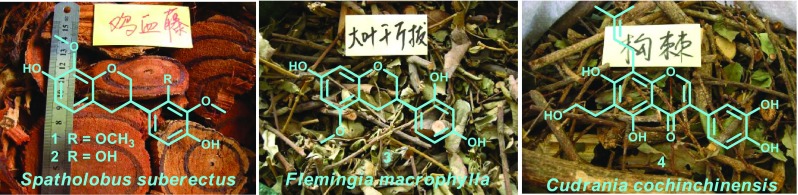

**Electronic supplementary material:**

The online version of this article (doi:10.1007/s13659-017-0121-2) contains supplementary material, which is available to authorized users.

## Introduction

**Fig. 1:**
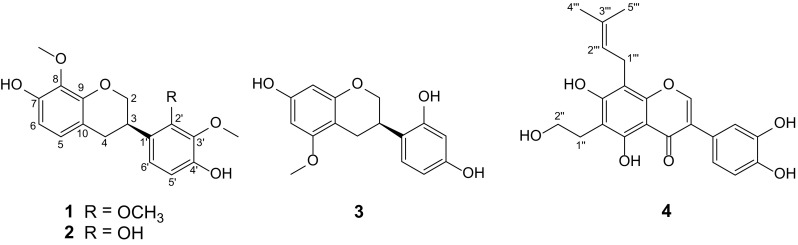
Structures of new compounds **1**–**4**

Isoflavonoids are a large group of secondary metabolites with diverse biological activities that occur widely in plants, which can be subdivided into isoflavones, isoflavans, isoflavanones, rotenoids, pterocarpans, etc. An overwhelming number of isoflavonoids reported come from the Leguminosae family, and some non-leguminous families such as Moraceae are also relatively abundant in isoflavonoids [1, 2]. Some isoflavans and prenylated isoflavones possess potent biological activities, especially in cytotoxicity [3–6], anti-inflammatory [7], and neuroprotective activity [8]. As part of a BioBioPha (http://www.chemlib.cn) objective to assemble a large-scale natural product library valuable in the discovery of new drug leads from nature [9–12], the phytochemical investigations on *Spatholobus suberectus* (Leguminosae), *Flemingia macrophylla* (Leguminosae), and *Cudrania cochinchinensis* (Moraceae) led to the isolation of three new isoflavans 7,4′-dihydroxy-8,2′,3′-trimethoxyisoflavan (**1**), 7,2′,4′-trihydroxy-8,3′-dimethoxyisoflavan (**2**), and 7,2′,4′-trihydroxy-5-methoxyisoflavan (**3**), and one new prenylated isoflavone namely cudraisoflavone M (**4**), respectively (Fig. 1). Herein we report the structure elucidation of new isoflavonoid and their cytotoxicity evaluation against five human cancer cell lines (HL-60, A-549, SMMC-7721, MCF-7, and SW-480).

## Results and Discussion

**Fig. 2:**
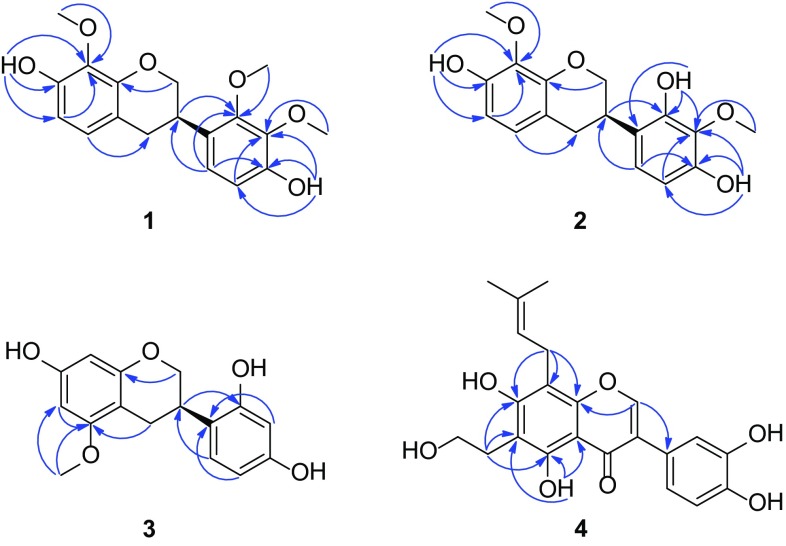
Key HMBC () correlations of **1**–**4**

Compound **1** was obtained as white amorphous powder, and its molecular formula was determined to be C_18_H_20_O_6_ from the positive HRESIMS at *m*/*z* 355.1163 [M+Na]^+^ (calcd. for C_18_H_20_O_6_Na, 355.1158) with nine degrees of unsaturation. The ^1^H NMR spectrum (Table 1) displayed five aliphatic proton signals due to two methylene groups [*δ*_H_ 2.86 (1H, ddd, *J* = 15.8, 5.3, 1.9 Hz) and 2.94 (1H, dd, *J* = 15.8, 11.3 Hz), 4.00 (1H, t, *J* = 10.5 Hz) and 4.37 (1H, ddd, *J* = 10.5, 3.7, 1.9 Hz)], and one methine [*δ*_H_ 3.53 (1H, dddd, *J* = 11.3, 10.5, 5.3, 3.7 Hz)], which were characteristic of an isoflavan [13]. The ^1^H NMR spectrum also exhibited two pairs of *ortho*-coupled aromatic doublets [*δ*_H_ 6.52 (1H, d, *J* = 8.3 Hz), 6.72 (1H, d, *J* = 8.3 Hz), 6.71 (1H, d, *J* = 8.5 Hz), and 6.75 (1H, d, *J* = 8.5 Hz)], three methoxy groups [*δ*_H_ 3.87, 3.92 and 3.93 (each 3H, s)], and two phenolic hydroxy protons [*δ*_H_ 5.66 and 5.71 (each 1H, s)]. The ^13^C NMR spectrum (Table 2) showed a total of 18 carbon signals, including 12 aromatic carbons for two phenyl units (rings A and B), the ring C carbons at *δ*_C_ 31.4 (d), 31.5 (t), 70.5 (t), and three methoxy carbons at *δ*_C_ 60.6, 60.8, 61.0 (each q). The above NMR spectroscopic features were very similar to those of isoduartin (=7,2′-dihydroxy-8,3′,4′-trimethoxyisoflavan) [14], except for an obvious downfield shift for H-5′ (Δ = +0.27 ppm), which hinted a different substituted pattern in ring B. The pattern can be determined as 4′-hydroxy-2′,3′-dimethoxy by the following HMBC correlations (Fig. 2): from 4′-OH (*δ*_H_ 5.71) to C-3′ (*δ*_C_ 139.8), C-4′ (*δ*_C_ 148.5) and C-5′ (*δ*_C_ 110.5); from H-3 (*δ*_H_ 3.53) and 2′-OCH_3_ (*δ*_H_ 3.87) to C-2′ (*δ*_C_ 150.8); and from 3′-OCH_3_ (*δ*_H_ 3.93) to C-3′. The particular downfield shifts (Δ ≈ +5 ppm) of aromatic methoxy carbons also implied the location of these groups. According to the empirical rule, the carbon signals of the methoxy groups with substituents in both *ortho* positions will appear at 60–62 ppm, while those sterically non-hindered at 55–57 ppm [15]. On the basis of the above analysis, the structure of **1** was established as 7,4′-dihydroxy-8,2′,3′-trimethoxyisoflavan.

**Table 1:** ^1^H NMR spectroscopic data of compounds **1**–**3**

No.	**1** ^a^	**2** ^a^	**3** ^b^
2-H_*eq*_	4.37 (ddd, 10.5, 3.7, 1.9)	4.43 (ddd, 10.2, 3.4, 2.0)	4.16 (ddd, 10.2, 3.1, 1.9)
2-H_*ax*_	4.00 (dd, 10.5, 10.5)	4.07 (dd, 10.2, 10.2)	3.86 (dd, 10.2, 10.2)
3	3.53 (dddd, 11.3, 10.5, 5.3, 3.7)	3.52 (dddd, 10.9, 10.2, 5.3, 3.4)	3.34 (dddd, 10.8, 10.2, 5.5, 3.1)
4-H_*ax*_	2.94 (dd, 15.8, 11.3)	3.01 (dd, 15.8, 10.9)	2.59 (dd, 16.3, 10.8)
4-H_*eq*_	2.86 (ddd, 15.8, 5.3, 1.9)	2.90 (ddd, 15.8, 5.3, 2.0)	2.76 (ddd, 16.3, 5.5, 1.9)
5	6.72 (d, 8.3)	6.72 (d, 8.3)	
6	6.52 (d, 8.3)	6.52 (d, 8.3)	5.99 (d, 2.1)
8			5.88 (d, 2.1)
3′			6.30 (d, 2.4)
5′	6.71 (d, 8.5)	6.47 (d, 8.5)	6.24 (dd, 8.3, 2.4)
6′	6.75 (d, 8.5)	6.74 (d, 8.5)	6.85 (d, 8.3)
7-OH	5.66 (s)	5.69 (s)	
2′-OH		5.72 (s)	
4′-OH	5.71 (s)	5.30 (br s)	
5-OCH_3_			3.74 (s)
8-OCH_3_	3.92 (s)	3.91 (s)	
2′-OCH_3_	3.87 (s)		
3′-OCH_3_	3.93 (s)	3.88 (s)	

^a^Measured in CDCl_3_ (*δ*_H_ 7.26 ppm)

^b^Measured in CD_3_OD (*δ*_H_ 3.30 ppm)

**Table 2:** ^13^C NMR spectroscopic data of compounds **1**–**3**

No.	**1** ^a^	**2** ^a^	**3** ^b^
2	70.5 (t)	69.8 (t)	71.0 (t)
3	31.4 (d)	31.8 (d)	32.6 (d)
4	31.5 (t)	30.3 (t)	26.1 (t)
5	124.3 (d)	124.3 (d)	160.1 (s)
6	107.0 (d)	106.9 (d)	92.3 (d)
7	147.5 (s)	147.4 (s)	157.7 (s)
8	134.8 (s)	134.7 (s)	96.5 (d)
9	147.1 (s)	147.1 (s)	156.9 (s)
10	115.3 (s)	115.3 (s)	104.1 (s)
1′	126.3 (s)	119.9 (s)	120.4 (s)
2′	150.8 (s)	147.1 (s)	157.2 (s)
3′	139.8 (s)	134.5 (s)	103.5 (d)
4′	148.5 (s)	147.4 (s)	157.9 (s)
5′	110.5 (d)	107.8 (d)	107.6 (d)
6′	122.0 (d)	122.7 (d)	128.8 (d)
5-OCH_3_			55.8 (q)
8-OCH_3_	61.0 (q)	60.9 (q)	
2′-OCH_3_	60.8 (q)		
3′-OCH_3_	60.6 (q)	61.2 (q)	

^a^Measured in CDCl_3_ (*δ*_C_ 77.0 ppm)

^b^Measured in CD_3_OD (*δ*_C_ 49.0 ppm)

Compound **2**, isolated as white amorphous powder, had a molecular formula of C_17_H_18_O_6_ by the positive HRESIMS at *m*/*z* 341.1002 [M+Na]^+^ (calcd. for C_17_H_18_O_6_Na, 341.1001). Comparison of the ^1^H and ^13^C NMR spectral data (Tables 1, 2) with those of **1** revealed that a methoxy group in the ring B was replaced by a hydroxy. From the HMBC correlations of the OH signal at *δ*_H_ 5.72 (1H, s) to C-1′ (*δ*_C_ 119.9), C-2′ (*δ*_C_ 147.1), and C-3′ (*δ*_C_ 134.5), we could conclude that the emerging hydroxy was positioned at C-2′ (Fig. 2). Hence, the structure of **2** was established as 7,2′,4′-trihydroxy-8,3′-dimethoxyisoflavan.

Compound **3** was isolated as white amorphous powder, and its molecular formula was determined to be C_16_H_16_O_5_ according to the positive HRESIMS at *m*/*z* 289.1075 [M+H]^+^ (calcd. for C_16_H_17_O_5_, 289.1076). Five diagnostic ring C proton signals [*δ*_H_ 2.59 (1H, dd, *J* = 16.3, 10.8 Hz), 2.76 (1H, ddd, *J* = 16.3, 5.5, 1.9 Hz), 3.34 (1H, dddd, *J* = 10.8, 10.2, 5.5, 3.1 Hz), 3.86 (1H, dd, *J* = 10.2, 10.2 Hz), and 4.16 (1H, ddd, *J* = 10.2, 3.1, 1.9 Hz)], an ABX-type aromatic proton system [*δ*_H_ 6.30 (1H, d, *J* = 2.4 Hz), 6.24 (1H, dd, *J* = 8.3, 2.4 Hz), and 6.85 (1H, d, *J* = 8.3 Hz)], two *meta*-coupled aromatic doublets [*δ*_H_ 5.88 (1H, d, *J* = 2.1 Hz) and 5.99 (1H, d, *J* = 2.1 Hz)], and one methoxy signal [*δ*_H_ 3.74 (3H, s)] were observed in the ^1^H NMR spectrum (Table 1), which revealed that **3** was a monomethyl ether derivative of 5,7,2′,4′-tetrahydroxyisoflavan. The HMBC correlations from the methoxy and H-4 proton signals to C-5 (*δ*_C_ 160.1) confirmed the methoxy group at C-5 (Fig. 2). Therefore, the structure of **3** was established as 7,2′,4′-trihydroxy-5-methoxyisoflavan.

Absolute configurations of the isoflavans (**1**–**3**) were established by comparison of the CD curves with those reported earlier for closely related analogues. All the CD spectra showed a negative Cotton effect in the transition region (215–240 nm), similar with those of 7-*O*-methylisomucronulatol, (3*R*)-isomucronulatol, and abruquinone L [16–19], which indicated the absolute configurations at C-3 of **1**–**3** being *R*-form. And optical rotation measurements were also taken to assign the absolute configuration. We carefully examined the specific rotation values of some 2′,3′,4′-trisubstituted isoflavans (Table 3), and the results suggested that the methoxy group substituted at C-2′ had a huge impact on the specific rotation, probably on account of the spatial proximity. For 2′-methoxyisoflavans, *R*-/*S*-form result in positive/negative specific rotation values, respectively, but for 2′-hydroxyisoflavans, *R*-form gives negative values [14, 17, 20–25]. Our current research results were consistent with the above empirical rule.

**Table 3:** Specific rotation values of some 2′,3′,4′-trisubstituted isoflavans

Compound	Substitution pattern							[*α*]_D_	References
	3	6	7	8	2′	3′	4′		
(−)-Duartin	*S*	–	OH	OMe	OMe	OH	OMe	−18.5 (CHCl_3_)	[20]
Mucronulatol	*S*	–	OH	–	OMe	OH	OMe	−18.5 (Me_2_CO)	[21]
Bryaflavan	*S*	OH	OH	–	OMe	OH	OMe	−17.3 (MeOH)	[22]
2′-*O*-Methylisomucronulatol	*R*	–	OH	–	OMe	OMe	OMe	+6.5 (CHCl_3_)	[23]
(+)-Duartin	*R*	–	OH	OMe	OMe	OH	OMe	+3.4 (CHCl_3_)	[14]
8,3′-Dihydroxyvestitol	*R*	–	OH	OH	OH	OH	OMe	−5.2 (MeOH)	[24]
Isomucronulatol	*R*	–	OH	–	OH	OMe	OMe	−19.4 (EtOH)	[25]
7-*O*-Methylisomucronulatol	*R*	–	OMe	–	OH	OMe	OMe	−11.0 (CHCl_3_)	[17]

Compound **4**, yellowish amorphous powder, had a molecular formula of C_22_H_22_O_7_ according to the positive HRESIMS at *m*/*z* 399.1440 [M+H]^+^ (calcd. for C_22_H_23_O_7_, 399.1444). The NMR spectra (Table 4) showed an olefinic signal (*δ*_H_ 8.23, 1H, s; *δ*_C_ 154.3, d) and a chelated hydroxy group at *δ*_H_ 13.48 (1H, s), characteristic of a 5-hydroxyisoflavone skeleton [8, 13]. In addition, an ABX-type aromatic proton system [*δ*_H_ 7.15 (1H, d, *J* = 2.0 Hz), 6.86 (1H, d, *J* = 8.2 Hz), and 6.94 (1H, dd, *J* = 8.2, 2.0 Hz)], a prenyl group [*δ*_H_ 1.63, 1.79 (each 3H, s), 3.45 (2H, d, *J* = 7.1 Hz), and 5.22 (1H, br t, *J* = 7.1 Hz)], and a hydroxyethyl moiety [*δ*_H_ 2.97, 3.90 (each 2H, t, *J* = 5.2 Hz)] were also detected in the ^1^H NMR spectrum. The ^13^C NMR spectrum displayed a total of 22 carbon resonances, including two methyls, three *sp* ^3^ methylenes, five *sp* ^2^ methines, and 12 *sp* ^2^ quaternary carbons. These spectroscopic features were very similar to those of cudraisoflavone L, which was recently isolated from the same genus and shared the same molecular formula with **4** [26]. Their structural difference was only due to the location of the prenyl group and the hydroxyethyl moiety. The hydroxyethyl unit was located at C-6 from the HMBC correlations of H-1″ (*δ*_H_ 2.97) and 5-OH (*δ*_H_ 13.48) to C-5 (*δ*_C_ 158.5) and C-6 (*δ*_C_ 111.2), while the prenyl group at C-8 by the correlations of H-1‴ (*δ*_H_ 3.45) and H-2 (*δ*_H_ 8.23) to C-9 (*δ*_C_ 154.6). Thus the structure of **4** was established and named as cudraisoflavone M. It is worth mentioning that cudraisoflavone M (**4**) was also obtained in our phytochemical investigation on the plant *Derris robusta* (Leguminosae).

**Table 4:** NMR spectroscopic data of cudraisoflavone M (**4**) in acetone-*d*_6_ (*δ*_H_ 2.04 ppm, *δ*_C_ 29.8 ppm)

No.	*δ*_H_	*δ*_C_	No.	*δ*_H_	*δ*_C_
2	8.23 (s)	154.3 (d)	4′		146.1 (s)
3		123.5 (s)	5′	6.86 (d, 8.2)	115.8 (d)
4		182.0 (s)	6′	6.94 (dd, 8.2, 2.0)	121.4 (d)
5		158.5 (s)	1′′	2.97 (t, 5.2)	26.1 (t)
6		111.2 (s)	2″	3.90 (t, 5.2)	63.7 (t)
7		161.8 (s)	1‴	3.45 (d, 7.1)	22.4 (t)
8		107.7 (s)	2‴	5.22 (br t, 7.1)	123.3 (d)
9		154.6 (s)	3‴		131.8 (s)
10		105.8 (s)	4‴	1.63 (s)	25.8 (q)
1′		123.8 (s)	5‴	1.79 (s)	17.9 (q)
2′	7.15 (d, 2.0)	117.2 (d)	5-OH	13.48 (s)	
3′		145.5 (s)			

Considering the potent cytotoxic activity of some isoflavonoids, the cytotoxicity of these new isoflavonoids (**1**–**4**) was evaluated against five human cancer cell lines (HL-60, A-549, SMMC-7721, MCF-7, and SW-480) using the MTS method. DDP (cisplatin) and paclitaxel were used as positive controls. The results showed that **1** and **4** exhibited weak cytotoxic activity (Table 5), while **2** and **3** were inactive (IC_50_ values >40 µM) for all cell lines.

**Table 5:** Cytotoxic activities of compounds **1**–**4** against five human cancer cell lines

Compd.	HL-60	A-549	SMMC-7721	MCF-7	SW-480
**1**	19.27	>40	19.11	39.72	>40
**4**	20.60	18.77	25.25	24.30	24.53
DDP	1.49	22.30	18.60	30.10	20.50
Paclitaxel	<0.008	<0.008	<0.008	<0.008	<0.008

## Experimental Section

### General Experimental Procedures

Optical rotations were measured on Jasco P-1020 automatic digital polarimeter. CD spectra were recorded on a Chirascan spectropolarimeter (Applied Photophysics, Leatherhead, Surrey, UK). UV data were obtained from HPLC online analysis. IR spectra were obtained on a Bruker Tensor-27 infrared spectrophotometer with KBr pellets. NMR spectra were carried out on a Bruker Avance III 600 or DRX-500 spectrometer with deuterated solvent signals used as internal standards. ESIMS and HRESIMS were measured using Agilent G6230 time-of-flight mass spectrometer. Preparative MPLC was performed on a Büchi apparatus equipped with Büchi fraction collector C-660, Büchi pump module C-605 and manager C-615. Silica gel (200–300 mesh, Qingdao Marine Chemical Inc., China), MCI gel CHP-20P (75–150 μm, Mitsubishi Chemical Corporation, Japan), Chromatorex C-18 (40–75 μm, Fuji Silysia Chemical Ltd., Japan) and Sephadex LH-20 (GE Healthcare Bio-Sciences AB, Uppsala, Sweden) were used for column chromatography. Fractions were monitored and analyzed using TLC, in combination with an Agilent 1200 series HPLC system equipped by an Extend-C18 column (5 μm, 4.6 × 150 mm).

### Plant Material and Isolation (Table 6)

The retention times (*t*_R_) of **1**–**4** on an analytical HPLC Extend-C18 column (20% → 100% MeOH in H_2_O over 8.0 min followed by 100% MeOH to 13.0 min, 1.0 mL/min, 25 °C) were 7.51, 6.85, 6.61 and 9.12 min, respectively.

**Table 6:**
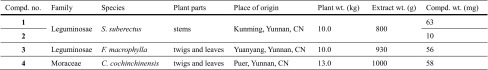
Plant material and isolation

#### 7,4′-Dihydroxy-8,2′,3′-trimethoxyisoflavan (**1**)

White amorphous powder; UV (MeOH) *λ*_max_: 228 (sh), 278 nm; [*α*]_D_^25^ +11.0 (*c* 0.20, MeOH); IR (KBr) *ν*_max_: 3396, 2944, 1602, 1497, 1464, 1292, 1198, 1170, 1097, 1068, 1040, 1010, 963 cm^−1^; ^1^H NMR data: see Table 1; ^13^C NMR data: see Table 2; ESIMS (pos.): *m*/*z* 355 [M+Na]^+^; HRESIMS (pos.): *m*/*z* 355.1163 [M+Na]^+^ (calcd. for C_18_H_20_O_6_Na, 355.1158).

#### 7,2′,4′-Trihydroxy-8,3′-dimethoxyisoflavan (**2**)

White amorphous powder; UV (MeOH) *λ*_max_: 229 (sh), 277 nm; [*α*]_D_^25^ −13.2 (*c* 0.20, MeOH); IR (KBr) *ν*_max_: 3404, 2929, 1619, 1509, 1470, 1328, 1184, 1091, 1058, 1037, 1001, 804 cm^−1^; ^1^H NMR data: see Table 1; ^13^C NMR data: see Table 2; ESIMS (pos.): *m*/*z* 341 [M+Na]^+^; HRESIMS (pos.): *m*/*z* 341.1002 [M+Na]^+^ (calcd. for C_17_H_18_O_6_Na, 341.1001).

#### 7,2′,4′-Trihydroxy-5-methoxyisoflavan (**3**)

White amorphous powder; UV (MeOH) *λ*_max_: 230 (sh), 279 nm; [*α*]_D_^25^ −24.0 (*c* 0.20, MeOH); IR (KBr) *ν*_max_: 3406, 2937, 1620, 1606, 1520, 1501, 1472, 1459, 1200, 1142, 1118, 1037, 974, 819 cm^−1^; ^1^H NMR data: see Table 1; ^13^C NMR data: see Table 2; ESIMS (pos.): *m*/*z* 289 [M+H]^+^; HRESIMS (pos.): *m*/*z* 289.1075 [M+H]^+^ (calcd. for C_16_H_17_O_5_, 289.1076).

#### Cudraisoflavone M (**4**)

Yellowish amorphous powder; UV (MeOH) *λ*_max_: 212, 270, 346 (sh) nm; IR (KBr) *ν*_max_: 3425, 2926, 1644, 1582, 1522, 1435, 1380, 1306, 1209, 1116, 1083, 1030 cm^−1^; ^1^H and ^13^C NMR data: see Table 4; ESIMS (pos.): *m*/*z* 399 [M+H]^+^; HRESIMS (pos.): *m*/*z* 399.1440 [M+H]^+^ (calcd. for C_22_H_23_O_7_, 399.1444).

### Cytotoxicity Assays

Five human tumor cell lines (HL-60, A-549, SMMC-7721, MCF-7, and SW-480) obtained from ATCC (Manassas, VA, USA) were used in the cytotoxicity assay. All cells were cultured in RPMI-1640 or DMEM medium (Hyclone, Logan, UT, USA), supplemented with 10% fetal bovine serum (Hyclone) at 37 °C in a humidified atmosphere containing 5% CO_2_. Cell viability was assessed by conducting colorimetric measurements of the amount of insoluble formazan formed in living cells based on the reduction of MTS (Sigma, St. Louis, MO, USA). Briefly, 100 μL of adherent cells were seeded into each well of a 96-well cell culture plate and allowed to adhere for 12 h before drug addition, while suspended cells were seeded just before drug addition, both with an initial density of 1 × 10^5^ cells/mL in 100 μL medium. Each cell line was exposed to the test compound at various concentrations in triplicate for 48 h, with cisplatin and paclitaxel as positive controls. After the incubation, 20 μL MTS and 100 μL medium was added to each well after removal of 100 μL medium, and the incubation continued for 2–4 h at 37 °C. The optical density was measured at 492 nm using a Multiskan FC plate reader (Thermo Scientific, USA). The IC_50_ value of each compound was calculated according to the Reed and Muench method.

## Electronic supplementary material

Below is the link to the electronic supplementary material.

Supplementary material 1 (DOCX 525 kb)
