# Supplementary material for: Polysubstituted Isoflavonoids from Spatholobus suberectus, Flemingia macrophylla, and Cudrania cochinchinensis
Source: Nat Prod Bioprospect. 2017 Jan 21;7(2):201–6. doi: 10.1007/s13659-017-0121-2 (PMC5397389; doi:10.1007/s13659-017-0121-2)

**Electronic Supplementary Material**

**Polysubstituted Isoflavonoids from** ***Spatholobus suberectus*,** ***Flemingia macrophylla*, and** ***Cudrania cochinchinensis***

Li-Xia Wang • Hai-Rong Zheng • Fu-Cai Ren • Tian-Ge Chen • Xiang-Mei Li • Xian-Jun Jiang • Fei Wang*

Structures of Compounds **1**–**4**

*To whom correspondence should be addressed.

E-mail: [f.wang@mail.biobiopha.com](mailto:f.wang@mail.biobiopha.com)

**Content list:**

**S1.** ^1^H NMR spectrum (600 MHz, CDCl_3_) of compound **1**.

**S2.** ^13^C NMR spectrum (150 MHz, CDCl_3_) of compound **1**.

**S3.** HMBC spectrum (600 MHz, CDCl_3_) of compound **1**.

**S4.** ^1^H NMR spectrum (600 MHz, CDCl_3_) of compound **2**.

**S5.** ^13^C NMR spectrum (150 MHz, CDCl_3_) of compound **2**.

**S6.** HMBC spectrum (600 MHz, CDCl_3_) of compound **2**.

**S7.** ^1^H NMR spectrum (500 MHz, CD_3_OD) of compound **3**.

**S8.** ^13^C NMR spectrum (100 MHz, CD_3_OD) of compound **3**.

**S9.** HMBC spectrum (500 MHz, CD_3_OD) of compound **3**.

**S10.** ^1^H NMR spectrum (600 MHz, acetone-*d*_6_) of compound **4**.

**S11.** ^13^C NMR spectrum (100 MHz, acetone-*d*_6_) of compound **4**.

**S12.** HMBC spectrum (600 MHz, acetone-*d*_6_) of compound **4**.

**S1.** ^1^H NMR spectrum (600 MHz, CDCl_3_) of compound **1**.


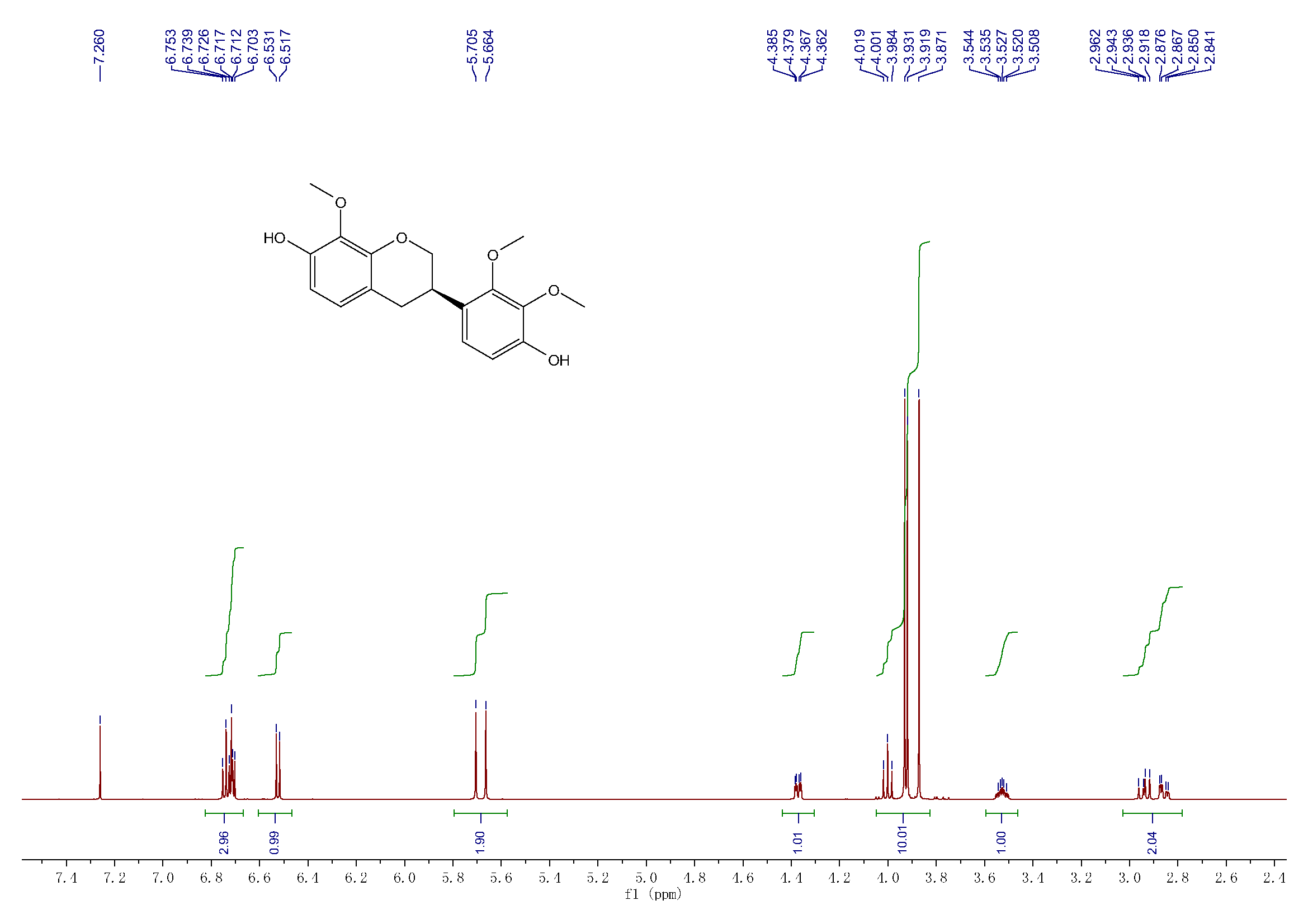


**S2.** ^13^C NMR spectrum (150 MHz, CDCl_3_) of compound **1**.


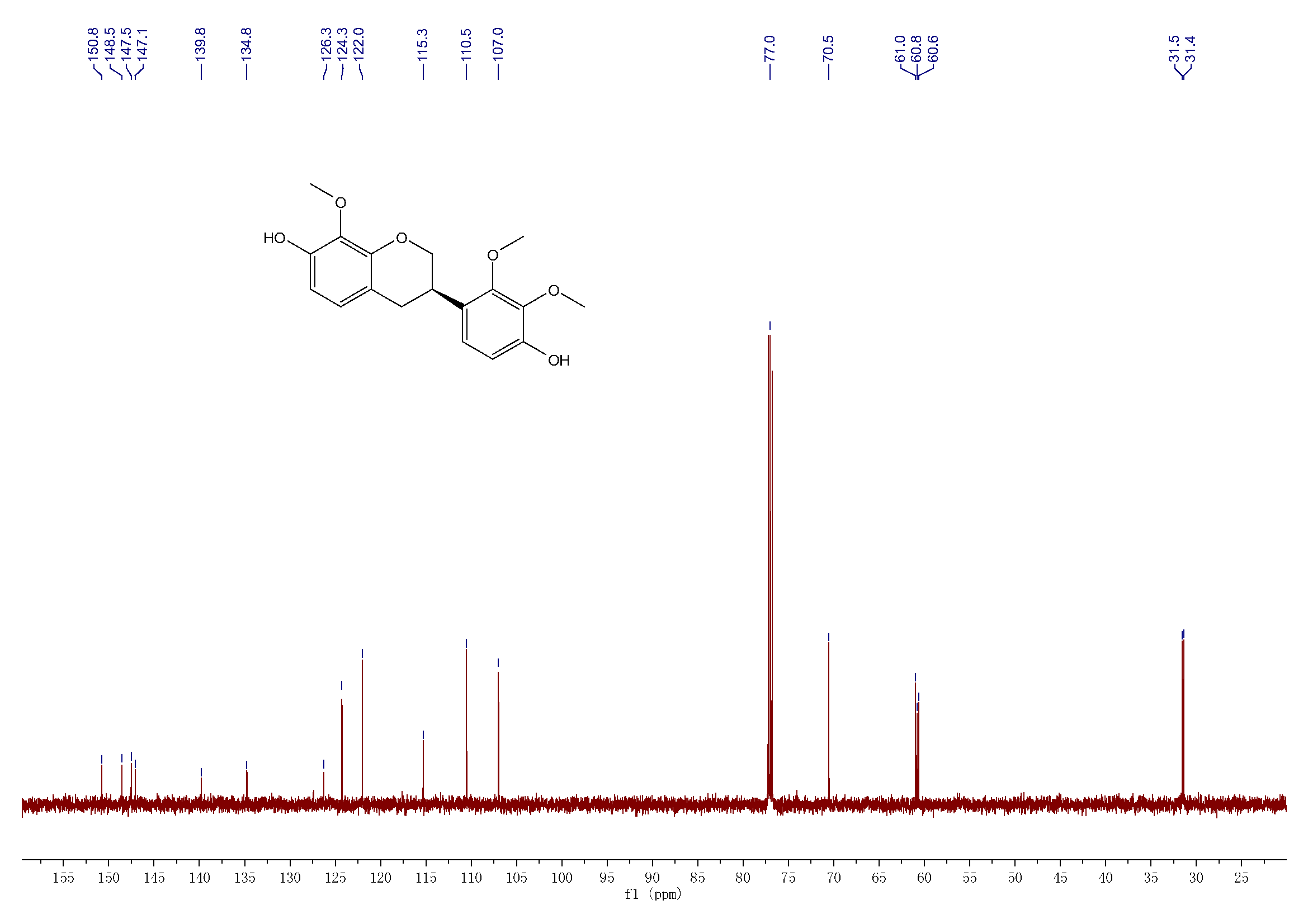


**S3.** HMBC spectrum (600 MHz, CDCl_3_) of compound **1**.


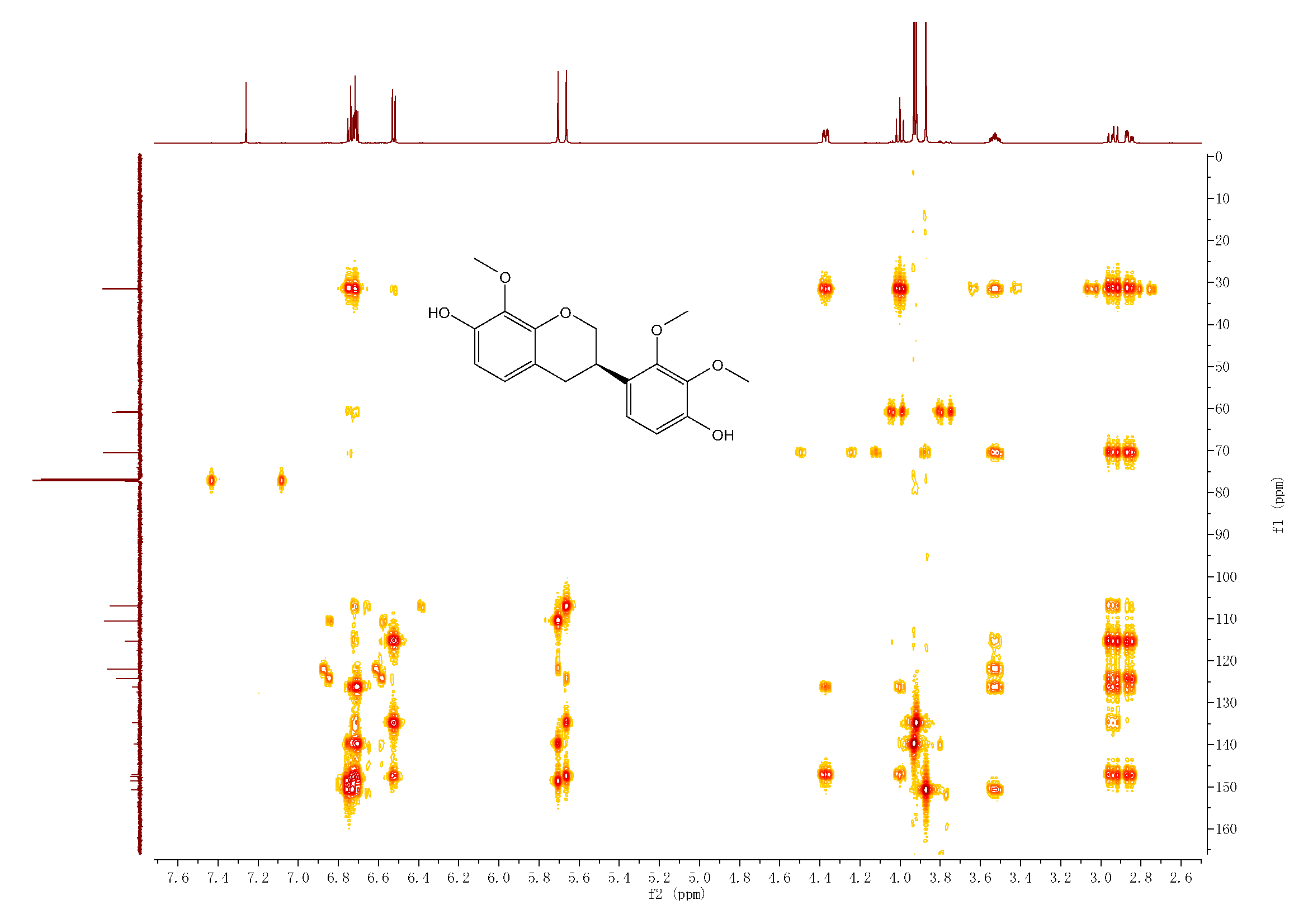


**S4.** ^1^H NMR spectrum (600 MHz, CDCl_3_) of compound **2**.


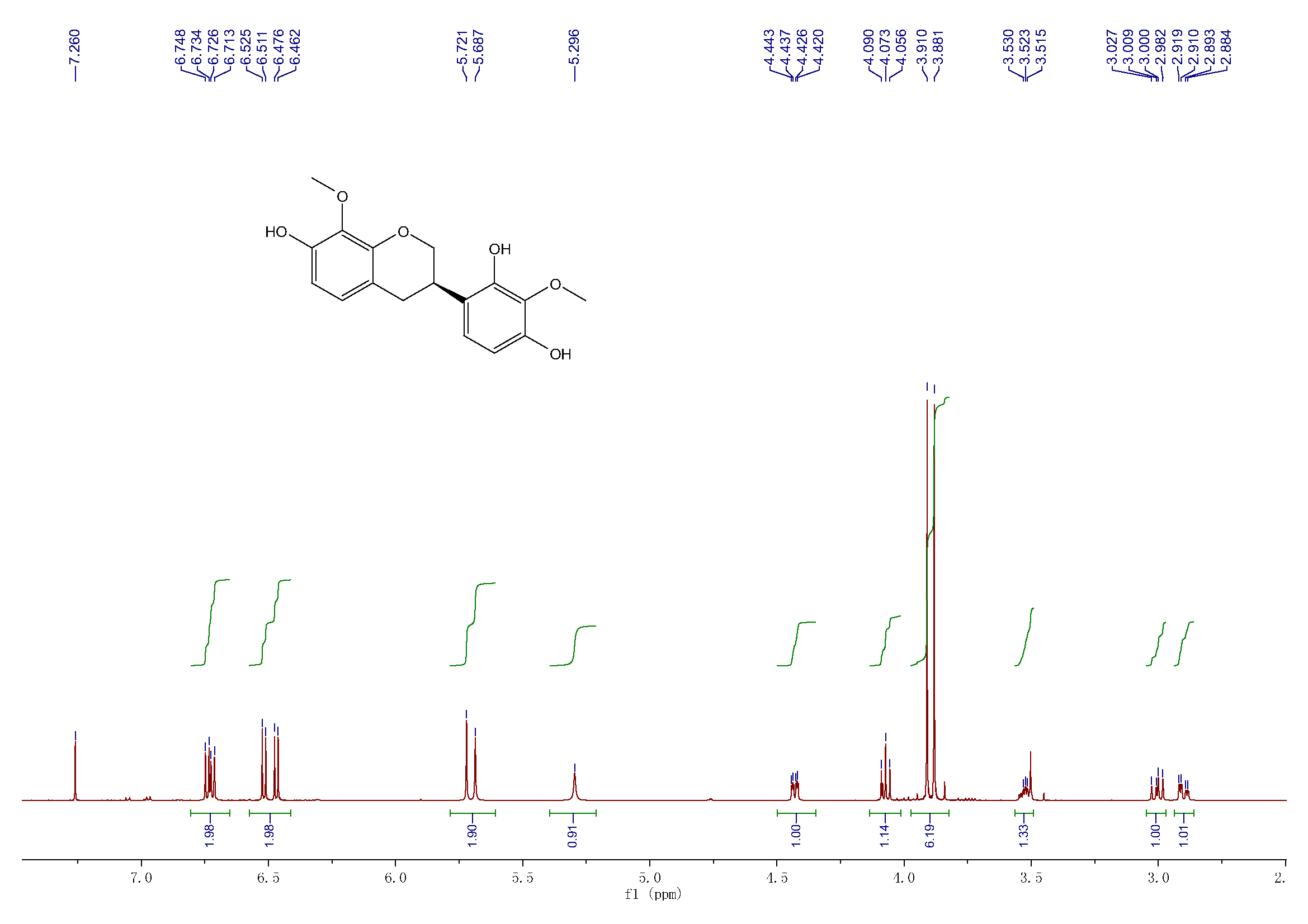


**S5.** ^13^C NMR spectrum (150 MHz, CDCl_3_) of compound **2**.


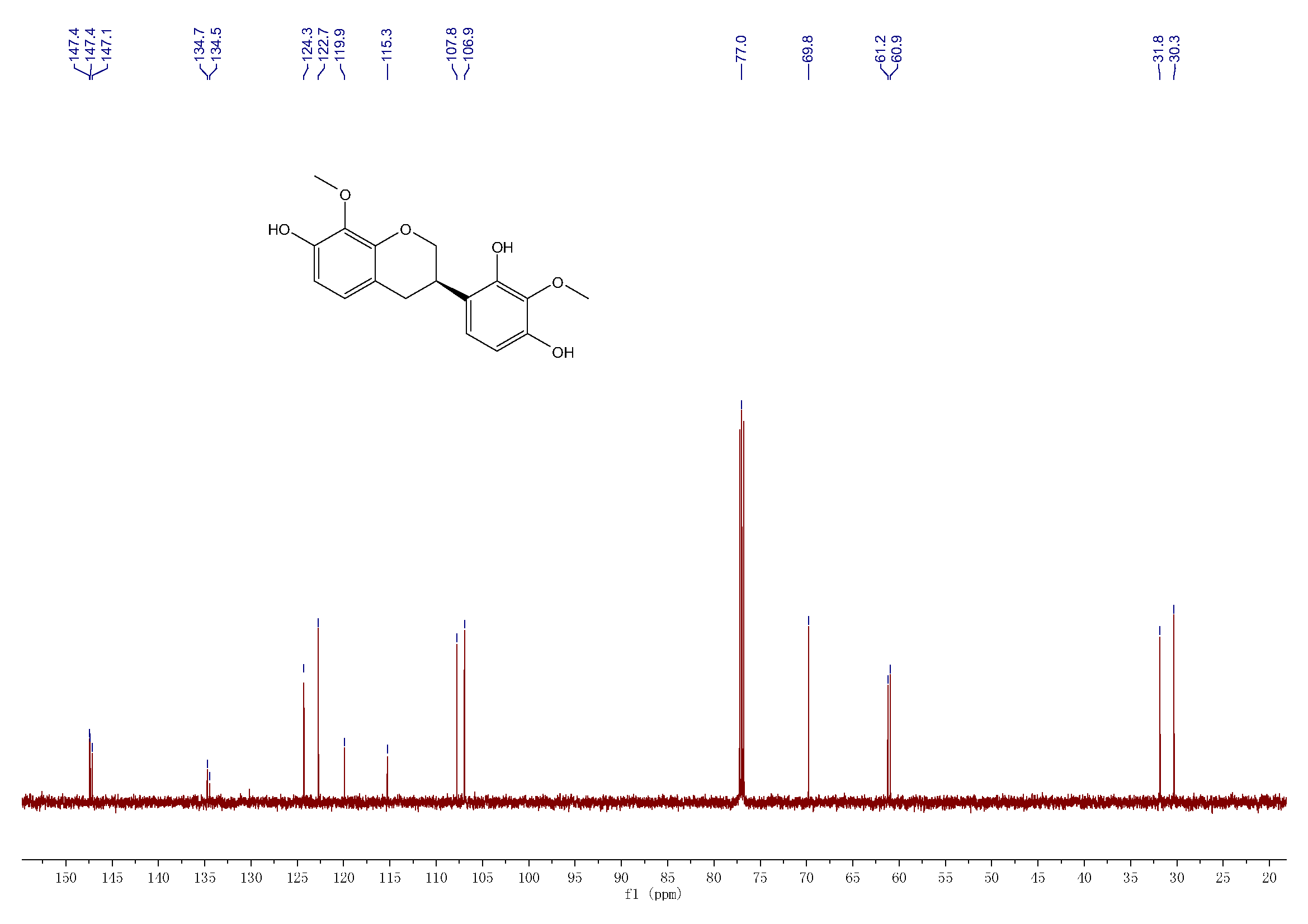


**S6.** HMBC spectrum (600 MHz, CDCl_3_) of compound **2**.


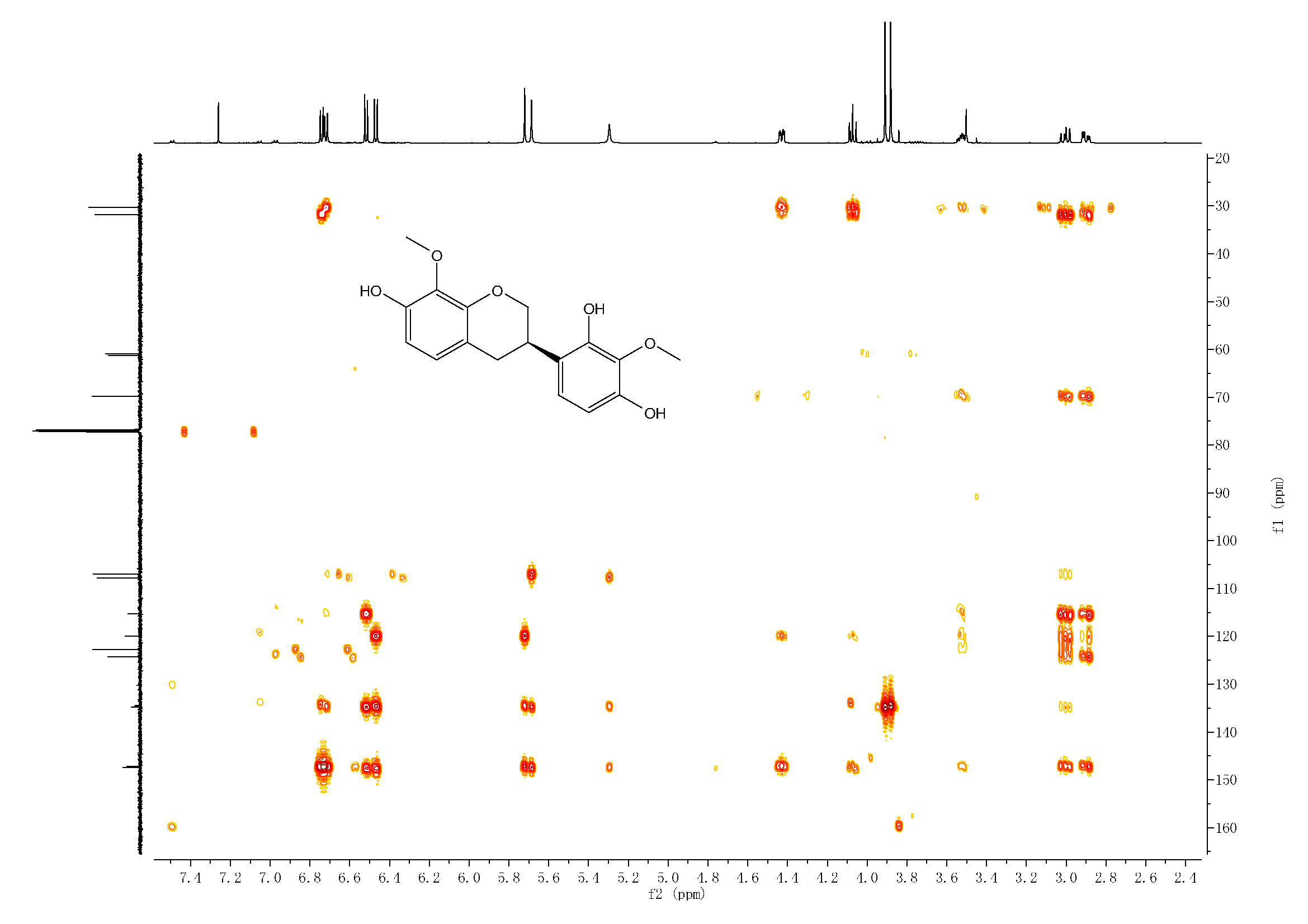


**S7.** ^1^H NMR spectrum (500 MHz, CD_3_OD) of compound **3**.


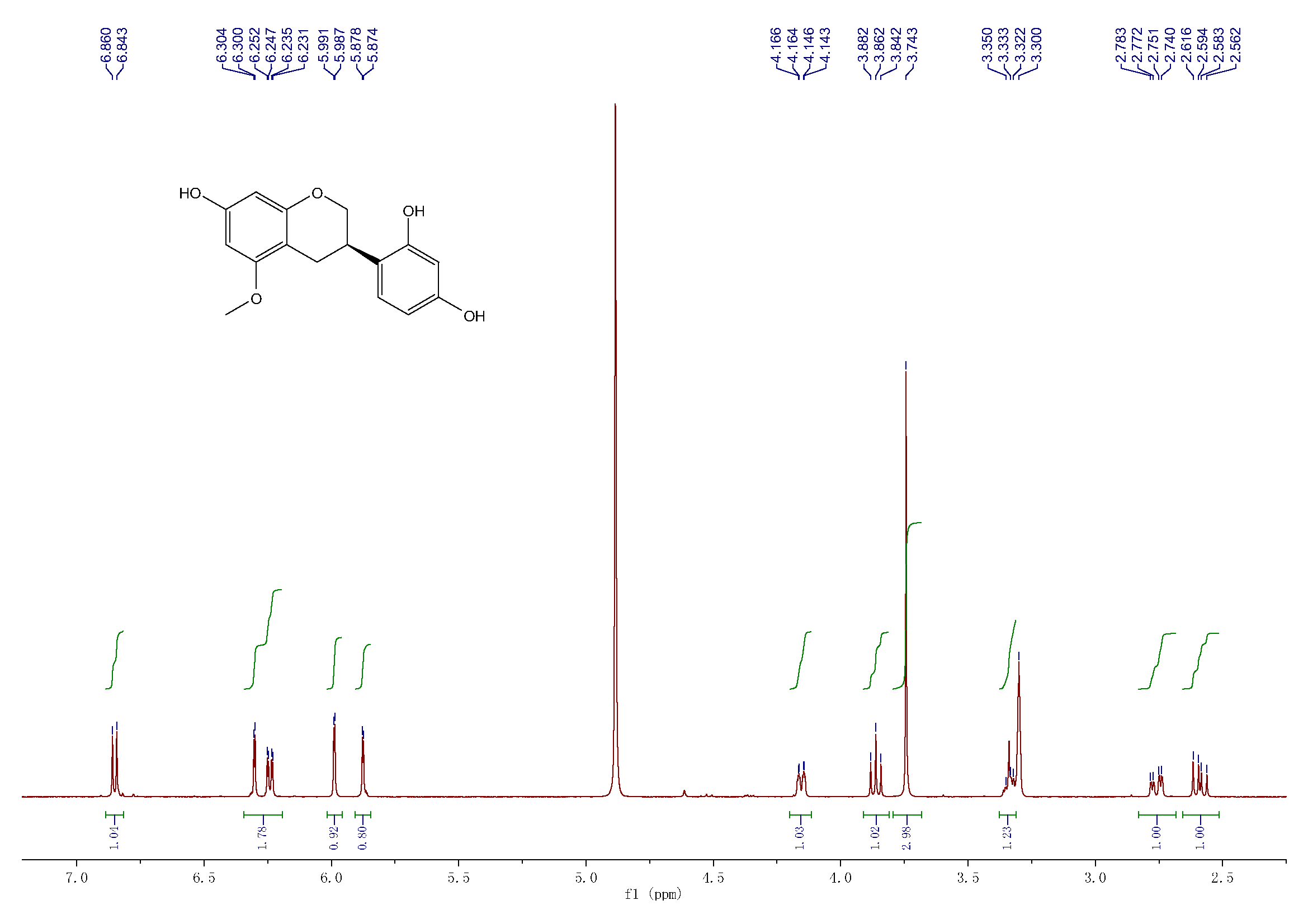


**S8.** ^13^C NMR spectrum (100 MHz, CD_3_OD) of compound **3**.


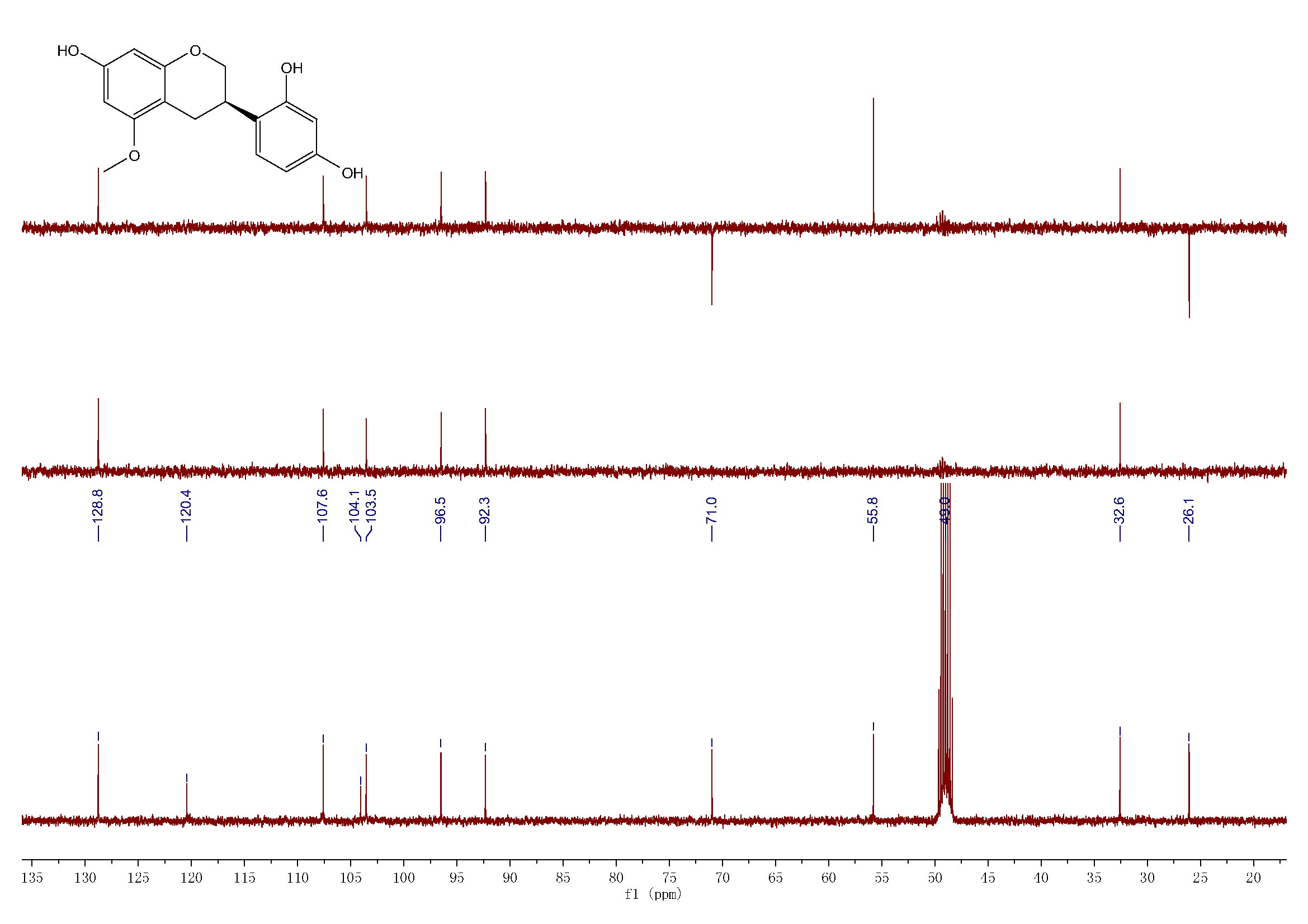


**S9.** HMBC spectrum (500 MHz, CD_3_OD) of compound **3**.


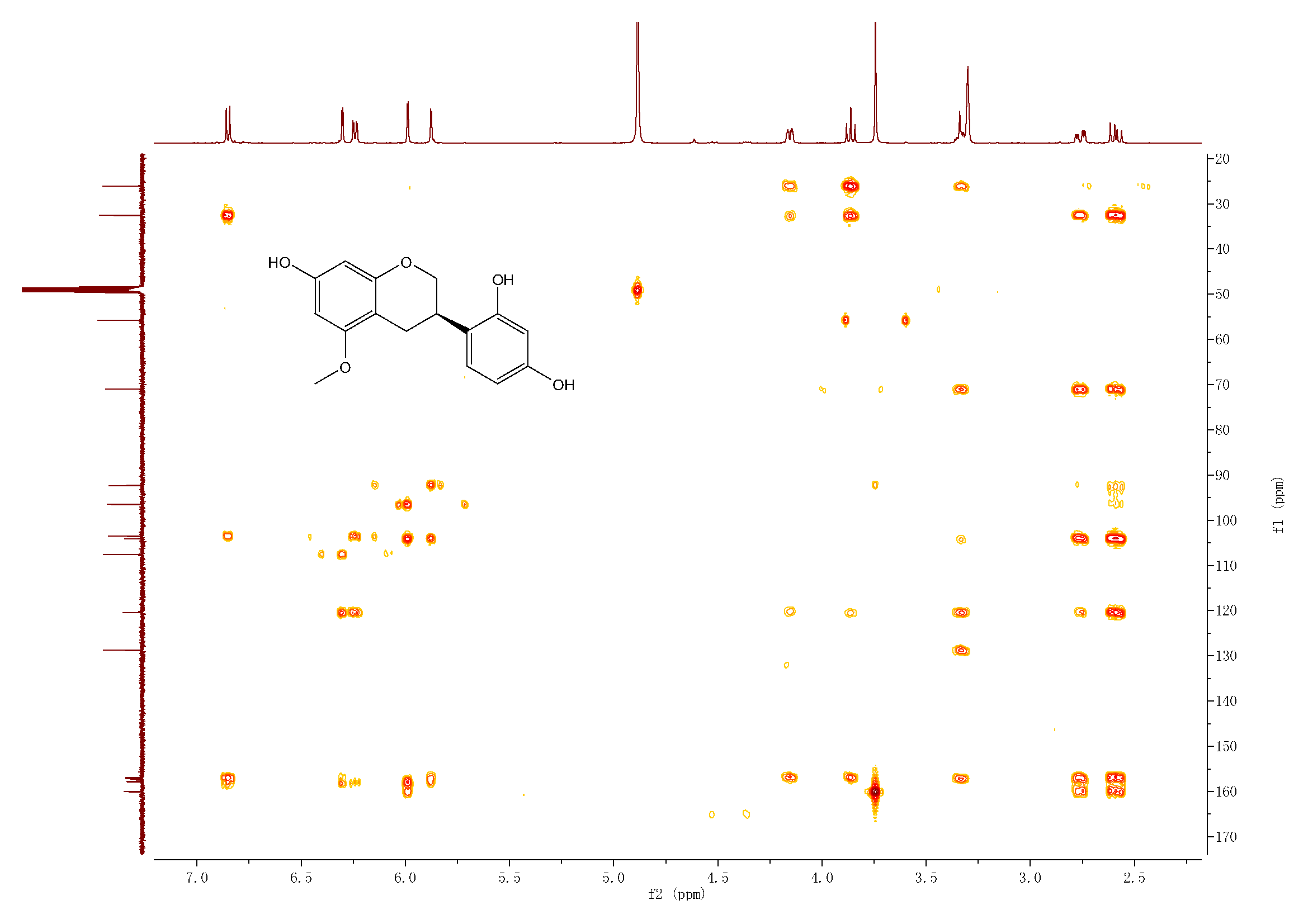


**S10.** ^1^H NMR spectrum (600 MHz, acetone-*d*_6_) of compound **4**.


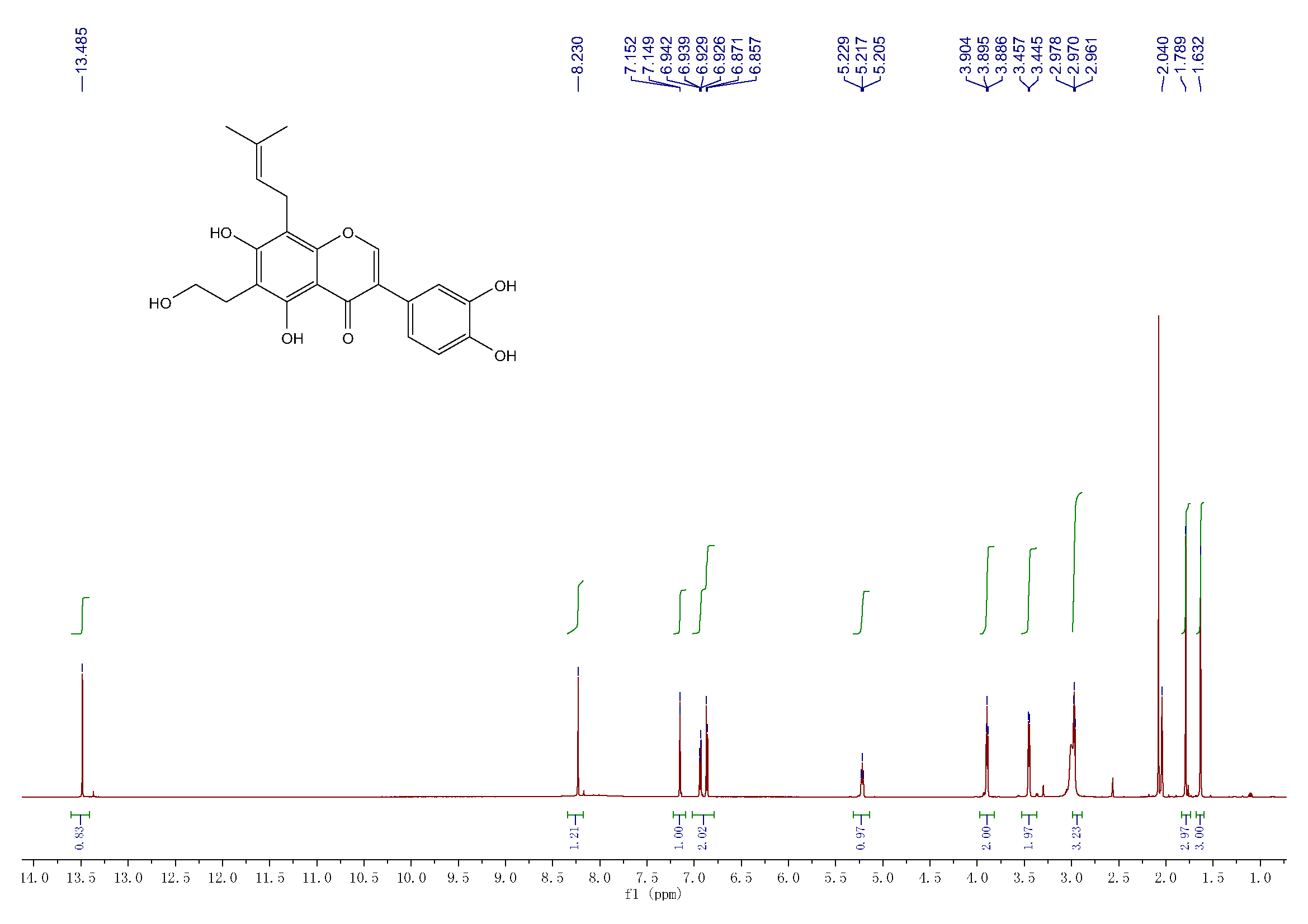


**S11.** ^13^C NMR spectrum (100 MHz, acetone-*d*_6_) of compound **4**.


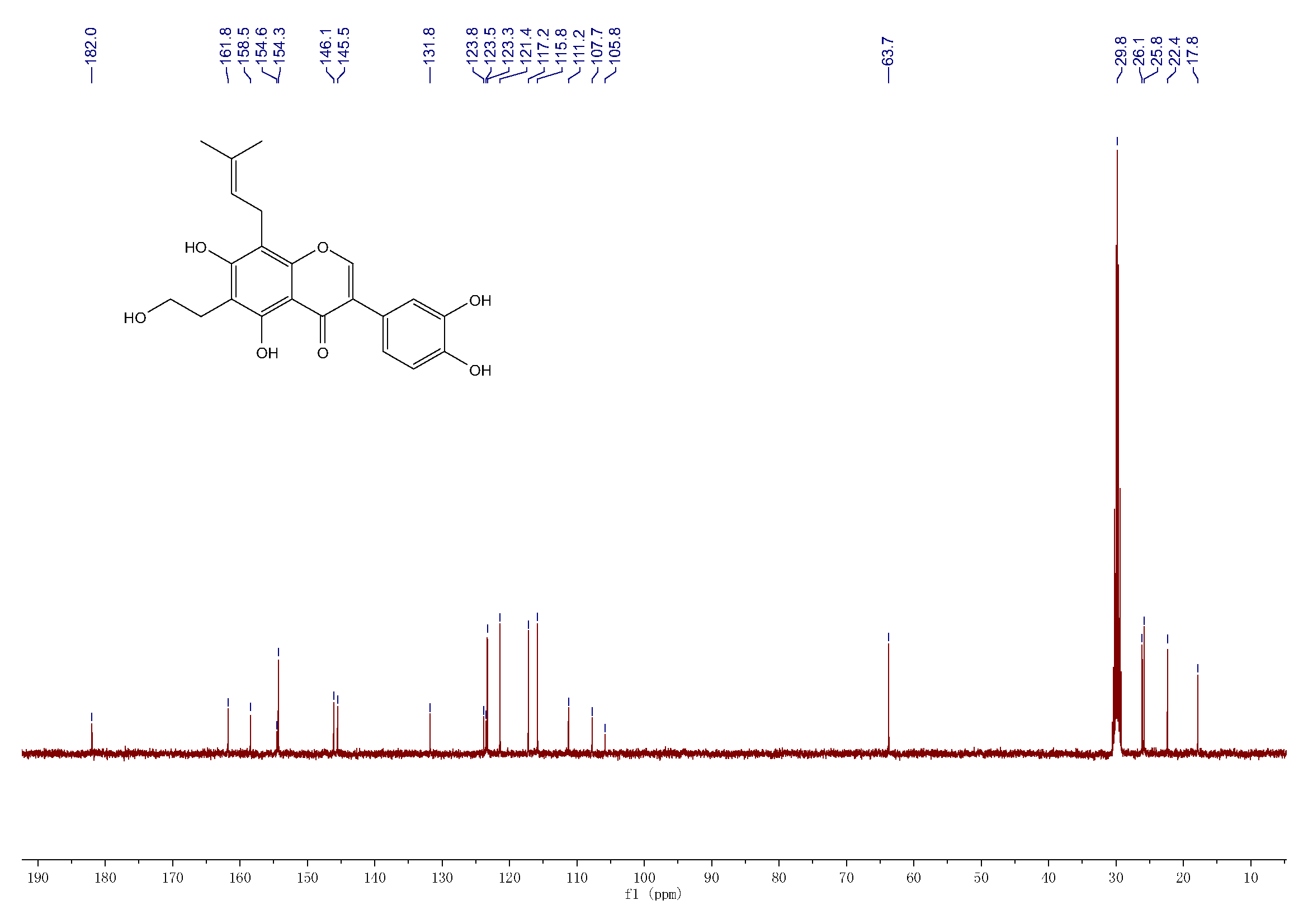


**S12.** HMBC spectrum (600 MHz, acetone-*d*_6_) of compound **4**.


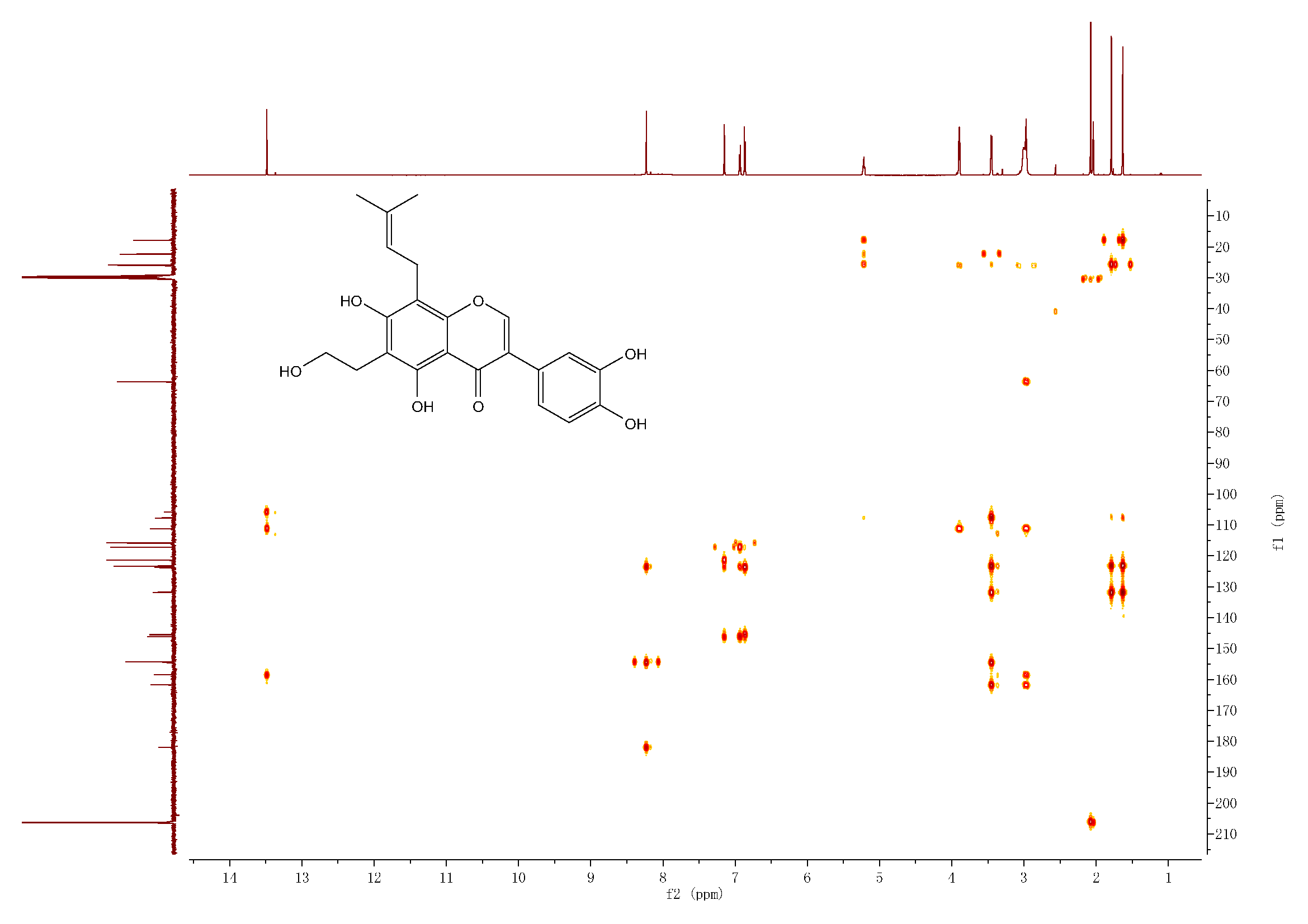

Supplement: Supplementary file 1 — Supplementary material 1 (DOCX 525 kb) [file 13659_2017_121_MOESM1_ESM.docx]
